# Methodological and reporting quality in laboratory studies of human eating behavior

**DOI:** 10.1016/j.appet.2018.02.008

**Published:** 2018-06-01

**Authors:** Eric Robinson, Kirsten E. Bevelander, Matt Field, Andrew Jones

**Affiliations:** a Institute of Psychology, Health & Society, University of Liverpool, L69 7ZA, UK; b Behavioural Science Institute, Communication Science, Radboud University, Nijmegen, The Netherlands

**Keywords:** Reporting, Methodology, Replication, Eating behavior, Nutrition

## Abstract

The methodological quality and reporting practices of laboratory studies of human eating behavior determine the validity and replicability of nutrition science. The aim of this research was to examine basic methodology and reporting practices in recent representative laboratory studies of human eating behavior. We examined laboratory studies of human eating behavior (N = 140 studies) published during 2016. Basic methodology (e.g., sample size, use of participant blinding) and reporting practices (e.g., information on participant characteristics) were assessed for each study. Some information relating to participant characteristics (e.g., age, gender) and study methodology (e.g., length of washout periods in within-subjects studies) were reported in the majority of studies. However, other aspects of study reporting, including participant eligibility criteria and how sample size was determined were frequently not reported. Studies often did not appear to standardize pre-test meal appetite or attempt to blind participants to study aims. The average sample size of studies was small (between-subjects design studies in particular) and the primary statistical analyses in a number of studies (24%) were reliant on very small sample sizes that would be likely to produce unreliable results. There are basic methodology and reporting practices in the laboratory study of human eating behavior that are sub-optimal and this is likely to be affecting the validity and replicability of research. Recommendations to address these issues are discussed.

## 1 Introduction

It is widely accepted that the way in which scientists design research and report their findings can be sub-optimal (Byrne et al., 2017, Pocock et al., 2004). Methodological weaknesses can affect the validity of study findings (Button et al., 2013, George et al., 2016) and inaccurate reporting of study methodology is likely to hamper replicability (Huwiler-Müntener et al., 2002, John et al., 2012). Alongside incentive structures in science to publish frequently and improper use of statistical analyses, sub-optimal methodological and reporting practices are likely to contribute to unreliable scientific findings (Ioannidis, 2005, Ioannidis, 2008, Simmons et al., 2011).

The validity of findings from nutritional epidemiology has been discussed of late (Ioannidis, 2016) and there is debate about whether the types of measures commonly used in such research (e.g., self-reported dietary instruments) are reliable (Dhurandhar et al., 2015, Subar et al., 2015). As yet, the quality of methodology and reporting practices of laboratory studies of human eating behavior have received little attention. Laboratory studies allow for controlled experimentation and identification of factors that predict and causally influence objectively measured energy intake (Blundell et al., 2010). Thus, the laboratory study of eating behavior plays an important role in nutrition science.

As with any scientific discipline, there are basic methodological design decisions that affect the quality of evidence that laboratory studies of eating behavior provide. Insufficient statistical power and very small sample sizes have been highlighted as a common problem in multiple disciplines (Button et al., 2013, Nord et al., 2017). For example, very small sample sizes are thought to increase the likelihood of false positive results and inflate effect size estimates (Szucs & Ioannidis, 2017). Likewise, taking measures to reduce the likelihood that ‘demand characteristics’ are compromising study findings is of importance; if participants are aware of the purpose or hypotheses of a study, this may influence their behavior and alter the results obtained in that study (Orne, 1962, Sharpe and Whelton, 2016). There are also methodological considerations specific to laboratory studies of human eating behavior. For example, standardizing appetite prior to the measurement of food intake is considered best practice (Livingstone et al., 2000) because hunger is likely to affect how much a person eats (Robinson et al., 2017, Sadoul et al., 2014).

Accurate reporting of methodology is important because it allows others to determine study quality, facilitates replication and ensures a study can inform evidence synthesis (i.e., inclusion in a systematic review and meta-analysis) (Simera et al., 2010). There are features of study reporting that are of obvious importance to many behavioral disciplines, such as accurate reporting of participant eligibility criteria (Schulz, Altman, & Moher, 2010), whereas other aspects of reporting are specific to the laboratory study of eating behavior. For example, the types of foods used to assess food intake and the length of washout period between test meals in within-subject design studies (Blundell et al., 2010). The aim of the present research was to examine the quality of basic methodological and reporting practices in recent representative laboratory studies of human eating behavior.

## 2 Method

### 2.1 Eligibility criteria

In order to capture current methodological and reporting practices of recent research studies we focused on studies published during 2016.^1^ Studies of human participants that were published during 2016 and used observational and/or experimental designs (within-subjects/‘cross over’, between-subjects/‘parallel arms’, or mixed designs) to examine objectively measured food intake (i.e., not participant self-reported food intake) in a laboratory setting were eligible for inclusion. Studies that were conducted in the field (e.g., a canteen), studies that measured food intake but did not report analyses on food intake as the dependent variable or studies that reported on validation of laboratory measurements of food intake were not eligible for inclusion.

### 2.2 Information sources and study selection

To examine recent and representative studies we used a journal driven approach, as in (Byrne et al., 2017). We identified peer reviewed academic journals that routinely publish laboratory studies of human eating behavior and searched all articles in these journals published during 2016. To identify journals we used a multi-stage expert consultation process during January–February 2017 involving 18 principal investigators of published laboratory studies of human eating behavior based in universities and research institutes across Europe, North America, Asia and Australasia. This process resulted in the identification of 24 journals. Examples of included journals are Appetite, Physiology & Behavior and the American Journal of Clinical Nutrition. For further information on the expert consultation process and the full list of journals, see S1.

### 2.3 Reporting and methodology coding

To identify basic reporting and methodological factors to be examined we consulted expert reports on best practice in laboratory eating behavior methodology (Blundell et al., 2010, Gibbons et al., 2014, Hill et al., 1995, Livingstone et al., 2000, Stubbs et al., 1998) and generalist reporting and methodology checklists for behavioral research (Higgins et al., 2011, Schulz et al., 2010, Welch et al., 2011).

### 2.4 Reporting

Coding instructions used are described in detail in S2. For each study we coded the study design (within-subjects, between-subjects, observational or mixed) and whether the following information was reported in the published manuscript or any accompanying online supplemental material:

• Summary data on participant gender (yes or no)
• Summary data on participant age (yes or no)
• Summary data on participant weight status (yes or no)
• Participant eligibility criteria used (yes or no)
• Information on how sample size was determined (yes or no)
• Information on how participants were allocated to experimental conditions (yes or no)
• Information on foods used to measure food intake (yes or no)
• The length of washout period between repeated measures of food intake (yes or no)
• Any statistical effect size information for food intake analyses (yes or no)

### 2.5 Methodology

We coded whether or not a study attempted to:

• Standardize appetite prior to measurement of food intake, e.g., participant fasting instructions or appetite standardized as part of laboratory visit (yes or no)
• Blind participants to study hypotheses, e.g., the use of a cover story or by ensuring experimental manipulations were concealed (yes or no)
• Measure participant awareness of study hypotheses, e.g., an end of study questionnaire/interview (yes or no)
• Whether studies had been registered, e.g., a clinical trials registry (yes or no)

### 2.6 Sample size

We examined the average (mean) number of participants per condition in within-subjects and between-subjects studies that primary statistical analyses were conducted on. For within-subject design studies this was the total number of participants that completed each experimental condition. For between-subject design studies this was calculated as being the mean number of participants per between-subject cell of the study. For example, a 2 × 2 between-subjects design with a total N = 100 contributed an average sample size of 25 participants per condition. We also used this information to assess the number of studies in which the primary statistical analysis was reliant on ‘very small’ sample sizes. Consistent with Simmons et al. (Simmons et al., 2011) for between-subject design studies we characterized ‘very small’ as N < 20 participants per condition. For within-subject design studies we characterized ‘very small’ as being N < 12 participants. For both study designs this number of participants would typically result in a study being grossly underpowered to detect a medium sized (*d* = 0.5) statistical difference (power (1 - β) < .33, α = .05, GPOWER 3.1) and inadequately powered to detect even a large sized (*d* = 0.8) statistical difference (power (1 - β) < .67, α = .05) between two experimental conditions for food intake.

### 2.7 Data collection process

Two authors independently performed the searches and were responsible for the evaluation of studies for inclusion, with disagreements resolved by discussion. All authors were responsible for data extraction. One author extracted data on sample size for all studies and a second author cross-checked a random sample of the extracted sample sizes (25%); there were no discrepancies. All other data were extracted by two authors independently so that inter-coder reliability could be formally assessed. Inter-coder reliability was consistently high, see S3. Any instances of coding disagreement between the two independent coders were resolved by a third author.

## 3 Results

### 3.1 Study selection

**Fig. 1:**
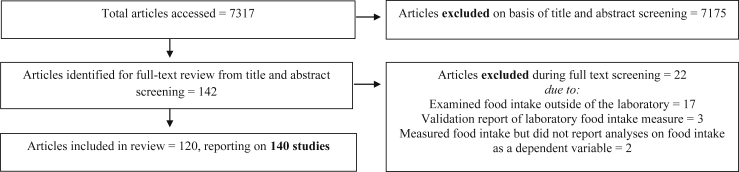
Study identification process.

One hundred and twenty articles reporting on a total of 140 laboratory studies of human eating behavior identified from searching 24 journals were eligible to be included in the review. See Fig. 1 for a flowchart of total articles identified, screened and reasons for exclusion. Of the 140 included studies, 63 (45.0%) adopted between-subjects designs, 51 (36.4%) within-subjects, 12 (8.6%) mixed designs, and 14 (10.0%) observational (i.e., no experimental manipulation) for their primary analyses on food intake. Coding for each study can be found online, see ‘data sharing’ section.

**Fig. 2:**
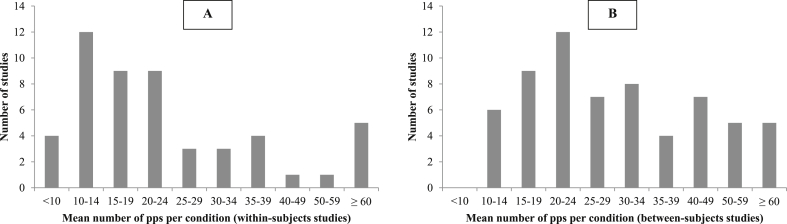
Number of participants per condition in within-subjects (A) and between subjects (B) studies.

### 3.2 Reporting

In total 137/140 (97.9%) of studies reported summary data on participant gender, 126/140 (90.0%) reported summary data on participant age and 104/140 (74.3%) on participant weight status. Information on participant eligibility criteria used was reported in 96/140 (68.6%) of studies and 54/140 (38.6%) reported how the recruited number of participants (sample size) had been determined. In studies that assigned participants to different experimental conditions (or order of conditions) 113/127 (89.0%) reported some information on how allocation was determined. The foods used to measure food intake were reported in (135/140, 96.4%) studies. In studies where a washout period between study sessions was used, 57/62 (91.9%) reported the length of washout period. Statistical effect size for any food intake analysis was reported in 85/140 (60.7%) of studies.

### 3.3 Methodology

Of the 140 studies, 99/140 (70.7%) of studies reported attempting to standardise appetite prior to measurement of food intake. Attempted blinding of participants to study hypotheses occurred in 75/140 studies (53.6%). Measurement of participant awareness of study hypotheses occurred in 34/140 studies (24.3%). Study registration was reported in 37/140 studies (26.4%).

### 3.4 Sample size

For within-subjects design studies (51 studies) the average number of participants per experimental condition ranged from 4–117 participants, with the mean and median number of participants per condition being 26.8 (SE = 3.3) and 20.0 respectively. In within-subject design studies, 21.6% (11/51) of studies had very small sample sizes (N < 12 per condition). For between-subject design studies (63 studies) the average number of participants per experimental condition ranged from 10.2–81.0 participants, with the mean and median number of participants per experimental condition being 31.5 (SE = 2.0) and 26.0 respectively. In between-subject design studies, 25.4% (16/63) had very small sample sizes (average N < 20 per condition) (see Fig. 2).

## 4 Discussion

We examined basic methodology and reporting practices of representative laboratory studies of human eating behavior published during 2016.

### 4.1 Quality of reporting

We found that some aspects of laboratory studies were consistently reported; the majority of studies (>90%) provided summary information on the gender and age of sampled participants, as well as the types of test foods used in studies and the length of time between sessions for studies in which food intake was repeatedly measured. However, other aspects of reporting were not consistent. One in four studies (25.7%) did not provide summary information about the weight status of the participants sampled and approximately one in three studies (31.4%) did not report information on participant eligibility criteria used. Because weight status is of relevance to eating behavior and laboratory studies often employ numerous participant exclusion criteria, studies failing to report such information result in an inadequate description of key sample characteristics. Although most studies (89.0%) reported some information on how participant allocation to experimental conditions was achieved, this information tended to be minimal (e.g., ‘randomly’). Some allocation methods can be presumed to be ‘random’ (e.g., allocation decided by the researcher or sequential allocation) but are not and therefore produce bias (Schulz & Grimes, 2002). More detailed descriptive reporting on allocation to experimental conditions would therefore be preferential. In addition, statistical effect size information was not consistently reported in studies. We took a conservative approach in which we coded for reporting of any statistical effect size information in any analyses relating to food intake (i.e. not just the primary analysis) and found that still 39.3% of studies reported no statistical effect sizes from analyses. This is problematic because such information is required to inform attempted replication and permits the effects observed in different studies to be formally compared, as well as synthesized to inform overall evaluations of evidence (e.g. meta-analysis) (Lakens, 2013). The amount of missing information in a published study may result in it not being possible to calculate statistical effect size and although study authors can be contacted, request for study information may often not be met (Selph, Ginsburg, & Chou, 2014).

### 4.2 Sample size

Most studies did not report how sample size was determined (61.4% of studies) and a sizeable minority of studies (24%) relied on very small sample sizes for their primary analyses; so small that they would be likely to produce unstable and therefore unreliable estimates of effect (Button et al., 2013). In addition, although some studies reported appropriate study sample sizes informed by power analyses, the typical sample sizes of within-subject and between-subject studies were relatively small. For example, many key influences on food intake, such as hunger and portion size yield ‘medium’ or smaller statistical effect sizes (Boyland et al., 2016, Cardi et al., 2015, Hollands et al., 2015, Robinson et al., 2014a, Sadoul et al., 2014, Vartanian et al., 2015). However, the average number of participants per condition in between-subject design studies observed here (mean = 32, median = 26) would result in a study being grossly underpowered to detect even a medium sized difference between two experimental conditions. It should be noted that we did not assess the statistical power of individual studies.^2^ Post-hoc calculation of statistical power for individual studies using ‘observed’ values is uninformative in this context because it will merely confirm that studies with ‘positive’ findings had a sufficient number of participants to detect the significant effect observed, whilst ‘null’ studies did not have a sufficient number of participants to be powered to detect a significant effect based on the observed values (see (Goodman & Berlin, 1994) for a detailed discussion). Estimating power for individual studies when the ‘true’ size of the studied effect has been identified (e.g., meta-analysis) would be more appropriate (Button et al., 2013, Nord et al., 2017). Nonetheless, our results suggest that sample size is a likely problem in the laboratory study of eating behavior; it is relatively common for studies to use sample sizes that may be too small to make confident inferences from.

### 4.3 Methodological practices

In assessing methodological practices of studies, we found that studies often did not report (29.3% of studies) any attempt to standardize appetite prior to measurement of food intake or state why standardizing was not used. Standardizing appetite is considered best practice because a lack of standardization would be likely to cause unaccounted for heterogeneity in appetite prior to measurement of food intake (Livingstone et al., 2000). A large proportion of studies also did not appear to attempt to blind participants to study hypotheses (46.4% of studies) or measure whether participants were aware of study hypotheses (75.7% of studies). It is therefore unclear to what extent participants were aware that food intake was being measured and/or the study hypotheses (that a measured or manipulated variable will be associated with food intake). Participant expectations about the hypotheses of a study (i.e., demand characteristics) can bias study results because they may cause participants to change their behavior. Thus, as is recognized in other areas of behavioral research, blinding participants to the study hypotheses and examining whether this blinding was successful addresses this threat to validity (Boutron et al., 2007, Sharpe and Whelton, 2016). There is evidence that self-reports of eating behavior can be biased by beliefs (Nix & Wengreen, 2017) and laboratory food intake has been shown to be biased by beliefs about the purpose of a study (Robinson et al., 2014b, Robinson et al., 2015). While it may be difficult to completely obscure from participants that food intake will be measured, we would argue that it is imperative that blinding to study hypotheses takes place and the extent to which this blinding is successful is measured and reported. Finally, we also examined how common it was for studies to be registered and found that a minority of studies were (26.4%). One of the reasons that study registration is likely to be beneficial is because if detailed procedural protocols and formal analysis plans are registered prior to data collection this should reduce selective reporting and publication bias (Munafò et al., 2017, Zarin and Tse, 2008). There are initiatives in other behavioral disciplines to encourage preregistration of research studies to become the norm (Nosek & Lakens, 2014) and we believe a similar change in practice in the laboratory study of eating behavior would improve the reliability of study findings.

### 4.4 Strengths and limitations

In the present work we evaluated a large number of recent and representative studies of laboratory human eating behavior. However, it should be noted that our article selection strategy will have resulted in the omission of studies published in journals that do not routinely publish laboratory eating behavior research and we examined published studies during 2016 only. Informed by expert reports we evaluated a number of basic reporting and methodological practices that we considered to be of importance to the laboratory study of human eating behavior. It was not feasible however, to evaluate all aspects of study design and reporting. For example, use of appropriate control/comparator groups in experimental research determines the extent to which individual studies can answer specific research questions. Thus, we acknowledge that although we examined many basic methodological and reporting features of studies, other aspects of study methodology and reporting are worthy of examination. Moreover, when evaluating aspects of study methodology, such as pre-session standardization of appetite, we inferred that the absence of any information denoted that a study did not standardize appetite. However, it is plausible that in some cases this occurred but was not reported, resulting in a reporting inaccuracy, rather than a methodological issue.

### 4.5 Recommendations

All researchers can improve the quality of research they are producing and our recommendations to improve the reporting and methodological design of laboratory studies of eating behavior are summarized in Table 1. However, for wide scale change in research practice to be achieved, ‘structural’ measures are likely to be necessary. For example, one approach is the development of a consensus statement on laboratory methods and an accompanying reporting checklist that is required at submission by journals that routinely publish such work. The development of a consensus statement would not be intended to hold back methodological innovation and as any field moves forward consensus statements need to be reconsidered. At present very few of the journals we surveyed required any form of reporting checklist at submission. The development of the CONSORT statement for clinical trials of health interventions was developed to improve quality of reporting, and reports suggest it has done so (Moher et al., 2001, Turner et al., 2012). Because existing reporting checklists such as CONSORT are not directly relevant to some reporting of laboratory research and also do not address key methodological issues in laboratory studies of eating behavior, the development of such a tool could be valuable but would require consensus from the field and a formal development and evaluation process. We also recommend that the methodological practices discussed here should be considered when evaluating quality of existing evidence. For example, if there are a sufficient number of published studies addressing a research question, then it will be possible to examine whether findings are consistent when the most methodologically sound studies are considered in isolation.

**Table 1:** Recommendations for laboratory studies of eating behaviour.

Recommendation	Benefit to field
Ensure appetite is standardised across participants prior to a laboratory test meal	Reduced undesirable variability in measurements of eating behavior

Minimise demand characteristics through blinding and measurement of participant awareness of study hypotheses	Better internal validity in studies

Conduct and report power analyses to inform sample size for primary analysis	Sufficiently powered studies

Examine and justify appropriateness of sample sizes used for any secondary or exploratory analyses	Fewer analyses reliant on very small sample sizes

Pre-register study protocol and detailed analysis strategy prior to data collection. For example, see: https://cos.io/rr/ Report any deviations from planned analysis strategy and report unplanned exploratory analyses as such	Increased transparency Fewer spurious post-hoc ‘discoveries’ Greater confidence in study findings
Report in detail all study methodology in manuscript or in supplemental materials Report all participant eligibility criteria used and sample demographics Detailed reporting of statistical results, including N of each analysis and effect size information	Facilitates replication Facilitates future evidence synthesis

## 5 Conclusions

There are basic methodology and reporting practices in the laboratory study of human eating behavior that are sub-optimal and this is likely to be affecting the validity and replicability of research.

## Data sharing

Coding criteria used are reported in full in the online supplemental materials and individual study coding is available as a date file at https://osf.io/n62h4/

## Funding

ER's salary is supported by the MRC (MR/N000218/1) and ESRC (ES/N00034X/1). ER has also received research funding from the American Beverage Association and Unilever.

## Conflicts of interest

The authors report no competing interests.

## Contributions

All authors contributed to the design and data collection process. ER drafted the manuscript and all authors provided critical revisions before approving the final version for publication.

## Footnotes

1 We opted to focus on a single year in order to make the total number of article reviewed feasible.

2 For example, within-subjects design studies that take multiple measurements of food intake under each experimental condition will have greater statistical power than within-subjects studies that only measure food intake once under each experimental condition. However, a single measurement of food intake under each experimental condition was typical among the studies we reviewed.
