# Supplementary material for: Methodological and reporting quality in laboratory studies of human eating behavior
Source: Appetite. 2018 Jun 1;125:486–91. doi: 10.1016/j.appet.2018.02.008 (PMC5890731; doi:10.1016/j.appet.2018.02.008)
Supplement: Supplementary material [file mmc1.docx]

**Online Supplemental Material: Methodological and Reporting Quality of Laboratory Studies of Human Eating Behavior**

**S1. Expert consultation process and full list of journals**

Through our own knowledge and online searches we devised an initial list of journals (N=16) that routinely publish observational and/or experimental laboratory studies that examine objectively measured food intake. We then consulted 13 investigators from Europe, North America, Asia and Australasia with a record of publishing such research. Each investigator was asked to review the initial list of journals and expand on it with any further journal recommendations. This consultation resulted in a further 4 journals being added to the inclusion list. We then consulted a further panel of 5 other investigators from Europe and North America with the updated list of included journals (N=20). Each investigator was asked to review the initial list of journals and expand on it with any further journal recommendations. This final consultation resulted in a further 4 journals being included.

Any journal that was suggested by 2 or more investigators during either stage of the expert consultation process was included in the final list of journals.

We searched articles from 24 journals:

American Journal of Clinical Nutrition

Appetite

British Journal of Nutrition

Eating Behaviors

European Journal of Clinical Nutrition

Food Quality and Preference

Frontiers in Eating Behavior

Health Psychology

International Journal of Behavioural Nutrition and Physical Activity

International Journal of Eating Disorders

International Journal of Obesity

Journal of Consumer Research

Journal of Experimental Psychology: Applied

Journal of Human Nutrition and Dietetics

Journal of Nutrition

Journal of the Academy of Nutrition and Dietetics

Nutrients

Nutrition

Obesity

Public Health Nutrition

Physiology & Behavior

PLoS One

Psychological Science

Psychology & Health

**S2. Coding criteria instructions**

**REPORTING CODING**

**Design of study:** Between-subjects experiment, within-subjects experiment, observational study, mixed design. ‘Between’ refers to analysis of different participants in different conditions. ‘Within’ refers to analysis of the same participants under different conditions. ‘Mixed’ refers to an analysis design that involves a combination of a between and within subject design. Observational is when an un-manipulated variable is used to predict food intake.

**Summary information reported about sample age:** Yes or no. Examples for ‘yes’ would include mean age or summary information that provides clear information about how old the majority of participants were (e.g. 80% of participants were 18-25 yrs old).

**Summary information reported about sample gender:** Yes or no. Examples for ‘yes’ would include numbers of males vs. female, or percentage of sample that are male/female.

**Summary information reported about sample weight / weight status:** Yes or no. Examples for ‘yes’ would include mean BMI, proportion of participants in different weight status categories.

**Information reported about participant inclusion/exclusion recruitment criteria:** Yes or no. Examples for ‘yes’ would include criteria that determined whether a participant was eligible to be recruited into the study or reporting that no exclusion/inclusion criteria were used. ‘No’ would constitute a lack of information about whether any inclusion/exclusion criteria were used. In some instances papers may report that some participants were excluded after data collection and prior to data analysis. This would not constitute information about participant inclusion/exclusion recruitment criteria.

**Justification of recruited sample size:** Yes or no. Is there any explanation or justification of how sample size was determined? Examples of ‘yes’ would include a power analysis stated as a-priori, sample size guidelines, sample size based on similar studies or pilot data. ‘No’ would constitute a lack of information about how sample size was determined. In some instances papers may report a power analysis based on the collected sample size post-hoc. This would not constitute information about justification of recruited sample size.

**Information about allocation of conditions in studies:** Yes or no. Examples of yes would include ‘random’ allocation to conditions, counterbalanced randomized order, use of Latin squares design.

**Reporting of food used:** Yes or no. Is there information about the type of food that was provided to participants?

**Reporting of washout for within-subjects (or mixed design studies including a within-subjects factor):** Yes or no. Is information about the length of time between within-subjects conditions reported?

**Reporting of statistical effect sizes for any food intake analyses?** Yes or no. Common examples of yes would include *d*, *f^2^*, partial eta or partial eta squared (η^2^), *r*, R^2^, beta coefficients.

**METHODOLOGICAL CODING**

**Pre-meal appetite standardization information:** Yes or no. Is there any explanation or justification of whether pre-meal standardization of appetite was attempted or apparent prior to measuring food consumption? Examples of ‘yes’ would include a fixed earlier meal as part of a laboratory visit, participant fasting instructions, or an explicit statement justifying why no standardization was used.

**Attempted concealment study aims:** Yes or no. Is there information reported that suggests the concealment of the study aims was attempted. Examples of ‘yes’ would include mention of a cover story to disguise the study aims or a manipulation that is described as being ‘blinded’ to participants (e.g. the effect of two ‘preload’ beverages that differ in energy density but they have been shown to be undetectable by appearance or sensory evaluation).

**Measurement of participant awareness of study aims:** Yes or no. Examples of ‘yes’ would include verbal questioning at the end of the study or a questionnaire in which participants are asked about their beliefs concerning the study aims.

**Study registration:** Is the study reported as having been registered? Yes or no.

**S3. Inter-coder reliability**

To examine inter-coder reliability Cohen’s kappa was calculated for each coding item, as well as percentage agreement. With one exception, Kappa ranged from 0.734 – 1 and percentage agreement ranged from 88 - 100%, indicating that inter-coder reliability for each coding item was high or perfect. The exception was a Kappa of 0.586 for ‘reporting of food used’ which would be considered ‘fair to good’. However, the percentage agreement between coders for this item was almost perfect (97.1% agreement: 136/140 cases). The lower Kappa value for this item is therefore likely to be due to the distribution of responses; nearly all studies were coded as ‘yes’ for reporting on study foods used. See McHugh (2012) for assumptions of the Kappa statistic and how these can result in Kappa excessively lowering apparent level of agreement.

McHugh ML. Interrater reliability: the kappa statistic. Biochemia Medica. 2012 Oct 15;22(3):276-82.
